# Elevated Uric Acid/Albumin Ratio Predicts Poor Coronary Collateral Circulation in Type 2 Diabetic Patients With Stable Coronary Artery Disease

**DOI:** 10.1155/jdr/9721061

**Published:** 2025-12-19

**Authors:** Lin Shuang Mao, Yi Xuan Wang, Yang Qi, Zhi Ran Yue, Feng Hua Ding, Xiao Qun Wang, Lin Lu, Wei Feng Shen, Ying Shen

**Affiliations:** ^1^ Department of Cardiovascular Medicine, Rui Jin Hospital, Shanghai Jiao Tong University School of Medicine, Shanghai, China, shsmu.edu.cn; ^2^ Institute of Cardiovascular Diseases, Shanghai Jiao Tong University School of Medicine, Shanghai, China, shsmu.edu.cn

**Keywords:** chronic total occlusion, coronary collateralization, stable coronary artery disease, type 2 diabetes, uric/albumin ratio

## Abstract

**Background:**

The uric acid/albumin ratio (UAR) has been shown to correlate with coronary disease severity and clinical outcome. This study investigated the predictive value of UAR on coronary collateralization (CC) in patients with type 2 diabetes mellitus (T2DM) and stable coronary artery disease.

**Methods:**

Serum levels of uric acid and albumin were determined and UAR was calculated in 1558 T2DM patients with chronic total occlusion of at least one major coronary artery. The degree of collaterals supplying to the distal occluded bed from the contra‐lateral vessel was graded by Rentrop scoring system.

**Results:**

Serum uric acid decreased and albumin increased gradually across Rentrop score 0 to 3, resulting in a higher UAR in patients with poor collaterals (Rentrop score 0 or 1) compared to those with good collaterals (Rentrop score 2 or 3) (9.74 [8.53–11.95] vs. 7.74 [6.61–8.84], *p* < 0.001). After adjusting for various confounders, elevated UAR was an independent factor for poor CC (adjusted OR 1.887, 95% CI 1.746–2.040, *p* < 0.001). UAR provided a better prediction for poor CC than uric acid and albumin alone (AUC 0.803 vs. 0.715 and 0.652; all *p* < 0.001). There existed an interaction between UAR and eGFR (*p* = 0.016); high UAR being associated with a greater risk (OR 2.253 vs. 1.776) and having a better predictive ability for poor CC (AUC 0.830 vs. 0.786; *p* = 0.041) in patients with eGFR ≥ 90 mL/min/1.73 m^2^ compared to those with eGFR < 90 mL/min/1.73 m^2^.

**Conclusions:**

Elevated UAR predicts poor coronary collateral circulation in T2DM patients with stable coronary disease especially when renal function is preserved. These findings may help physicians better identify high‐risk patients and guide individualized management strategies.

**Trial Registration:**

ClinicalTrials.gov identifier: NCT06054126

## 1. Introduction

Coronary collateral formation is an important protective adaptation against severe myocardium ischemia. Previous studies have shown that robust collaterals could reduce ischemic area, preserve left ventricular function, and improve the prognosis of patients with critical coronary stenosis [1, 2]. The formation of coronary collaterals is driven by a complex mechanism involving perfusion pressure, inflammatory cytokines, and endothelial function [3]. Abundant evidence suggests that coronary collateralization (CC) is impaired in patients with type 2 diabetes mellitus (T2DM), which may be attributed to several mechanisms. These include diffuse coronary atherosclerosis and microvascular dysfunction, chronic low‐grade inflammation, oxidative stress, and imbalances in pro‐ and anti‐angiogenic mediators [4–6]. Endothelial dysfunction is common in T2DM due to hyperglycemia and insulin resistance, which may critically limit arteriogenesis and angiogenesis. As a result, patients with T2DM tend to exhibit poorer collateral development, which in turn worsens ischemic burden and clinical outcomes. Coronary chronic total occlusion (CTO) is often caused by a heavy atherosclerotic plaque burden within the artery and was considered as a fundamental precondition for the spontaneous occurrence of collateral recruitment. Treatment decisions for CTO, including the need for percutaneous intervention, rely heavily on the degree of collateral status. Therefore, identifying reliable and accessible predictors of collateral status is particularly valuable in T2DM patients.

Circulating uric acid is the final product of purine nucleotide catabolism, and albumin is a negative acute‐phase reactant. Numerous studies have shown that elevated uric acid or reduced albumin levels or both are associated with the occurrence and severity of cardiovascular disease as well as increased morbidity and poor prognosis in these patients [7–9]. The uric acid/albumin ratio (UAR) by integrating these two measurements has emerged as a novel composite biomarker of systemic oxidative and inflammatory status [10–12]. Chronic hyperglycemia in diabetic patients activates low‐grade inflammation, oxidative stress, and endothelial dysfunction, all of which contribute to impaired angiogenesis and arteriogenesis. Elevated UAR has been linked to adverse cardiovascular outcomes and may reflect an unfavorable vascular environment that limits the development of adequate collateral vessels in diabetic patients. Recently, a few studies showed that UAR was related to collateral development in patients with coronary artery disease [13, 14], but none has been focusing on diabetic population. Given the known impairment in collateral formation and worse cardiovascular prognosis in diabetic patients, this population represents a clinically important yet understudied subgroup. Understanding whether UAR can serve as a predictive biomarker for CC in this context may aid in early risk stratification and guide management decisions, including revascularization strategies. As a result, this study aimed to determine the predictive ability of UAR for angiographic CC among a large number of T2DM patients who had stable coronary artery disease along with chronic total occlusion.

## 2. Methods

### 2.1. Study Population

**Figure 1:**
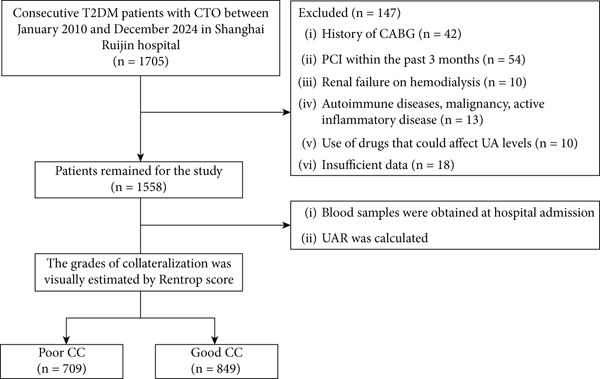
Flowchart of the study population. T2DM, type 2 diabetes mellitus; CTO, chronic total occlusion; CABG, coronary artery bypass grafting; PCI, percutaneous coronary intervention; UAR, uric acid‐to‐albumin ratio; CC, coronary collateralization.

The study cohort was retrospectively collected from January 2010 to December 2024. The study protocol and ethical approval for the retrospective analysis were registered in 2023. This registration ensures that the conduct of the study complies with ethical standards for retrospective data analyses. A cohort comprising 1705 consecutive patients, who were diagnosed with T2DM and stable coronary artery disease from January 2010 to December 2024, was enrolled in this study. All participants were over 18 years of age and had at least one major coronary artery showing 100% angiographic occlusion for over 3 months. The duration of occlusion was estimated based on clinical history including onset of stable symptoms or prior myocardial infarction, previous coronary angiograms or documented medical records. The exclusion criteria for this study were as follows: previous history of coronary artery bypass grafting, percutaneous coronary intervention within the past 3 months, hemodialysis, autoimmune disease, malignancy, active inflammatory disease defined as a clinical diagnosis of acute or chronic inflammation, use of drugs that could affect uric acid metabolism, or missing key clinical or laboratory data necessary for analysis, including serum uric acid, albumin, estimated glomerular filtration rate (eGFR), or angiographic collateral grading. After applying these criteria, 147 patients were excluded, and finally, 1558 T2DM patients with CTO were included in the final analysis (Figure 1). The protocol was approved by the Institutional Review Board of Rui Jin Hospital, Shanghai Jiao Tong University School of Medicine. We obtained written informed consent from all patients and carried out the study according to the Helsinki Declaration.

Demographic data, medical history, and risk factors for coronary artery disease were collected from the Inpatient Medical Record Management Systems. Upon admission, blood samples were obtained, and serum levels of uric acid, albumin, creatinine, aspartate aminotransferase, alanine aminotransferase, total cholesterol, low‐density lipoprotein cholesterol (LDL‐C), and high‐density lipoprotein cholesterol (HDL‐C) were measured using standard laboratory techniques. Uric acid was expressed in *μ*mol/L, and albumin in g/L. UAR was calculated by dividing the serum uric acid concentration (*μ*mol/L) by the serum albumin concentration (g/L). eGFR was determined using the Chronic Kidney Disease Epidemiology Collaboration (CKD‐EPI) equation: eGFR = 141 × min (creatinine/*к*, 1)^*α*^ × max (creatinine/*к*, 1)^−1.209^ × 0.993^age^ × 1.018 [if female], where к is 0.7 for females and 0.9 for males, *α* is − 0.329 for females and − 0.411 for males, min indicates the minimum of creatinine/к, or 1, and max indicates the maximum of creatinine/к, or 1 [15]. Left ventricular ejection fraction was assessed by two‐dimensional echocardiography according to the modified Simpson’s rule.

T2DM was diagnosed as fasting blood glucose ≥ 7.1 mmol/L, 2 h postprandial plasma glucose level ≥ 11.1 mmol/L, glycosylated hemoglobin A1c (HbA1c) ≥ 6.5%, or use of anti‐diabetic medication [16]. Hypertension was defined as systolic blood pressure ≥ 140 mmHg, diastolic blood pressure ≥ 90 mmHg, or taking anti‐hypertensive medications [17]. Hypercholesterolemia was defined as total cholesterol exceeding 200 mg/dL or using lipid‐lowering agents [18]. Smokers were defined as patients who smoked at least one cigarette daily in the previous 6 months [19]. An eGFR of ≥ 90 mL/min/1.73 m^2^ was considered as preserved renal function, whereas an eGFR of 89–60, 59–30, or < 30 mL/min/1.73 m^2^ was regarded as mild, moderate, or severe renal insufficiency, respectively.

### 2.2. Angiographic Procedure and Collateral Grading

Coronary angiography was performed with standard Judkins technique using a 6F catheter via the radial or femoral approach. Two experienced interventional cardiologists evaluated all angiographic images, and any disagreement was resolved by a third reviewer. The severity of coronary artery diseases was determined by the number of significant diseased coronary arteries (≥ 50% luminal diameter narrowing in a major epicardial coronary artery). Left main coronary stenosis ≥ 50% was regarded as two‐vessel disease. The degree of coronary collaterals was assessed using Rentrop scoring system, which is a semi‐quantitative method commonly used to grade collateral vessel filling. The scores are defined as follows [20]; 0 = no visible filling of any collateral vessels, 1 = filling of the collateral vessels without visibility of epicardial regions, 2 = filling of the collateral vessels with partial reaching the epicardial regions, and 3 = complete filling of the major epicardial artery by collateral vessels. The highest Rentrop score was selected for analysis when there were multiple CTO. Rentrop score 0 or 1 was classified as poor collateralization while 2 or 3 was regarded as good collateralization as in previous studies [1, 21].

### 2.3. Statistical Analysis

Continuous variables were expressed as mean ± standard deviations (SD) or median (interquartile range, IQR) depending on their distribution. The normality of continuous variables was assessed using the Shapiro–Wilk test and visual inspection of histograms. For normally distributed variables, Student’s *t* test or one‐way analysis of variance (ANOVA) was used to compare differences between groups, otherwise, the nonparametric Mann–Whitney *U* test was applied. Categorical variables were presented as an absolute number with percentage and Chi‐square test was used for comparison between groups. Spearman’s rho tests were applied to determine the correlation of uric acid, albumin, and UAR with Rentrop score. Multivariate logistic regression models were constructed to determine the independent factors for poor CC. Because UAR is derived from serum uric acid and albumin levels, uric acid and albumin were not included simultaneously with UAR in the same regression models to avoid multicollinearity and overadjustment. The linearity of continuous variables with the logit of the dependent variable was assessed using the Box‐Tidwell procedure, and multicollinearity was evaluated through variance inflation factors (VIFs). No significant violations were detected. Receiver operating characteristic (ROC) analysis was made to evaluate the predictive ability of uric acid, albumin and UAR on poor CC. Additionally, the Youden index was utilized to establish the optimal cutoff point that maximized both sensitivity and specificity for these predictive markers. DeLong test was used to compare the area under the ROC curve (AUC). All statistical analyses were performed using SPSS version 26.0 and MedCalc software version 20.0.22. A two‐tailed *p* value < 0.05 was considered statistically significant.

## 3. Results

### 3.1. Clinical Characteristics of the Study Population

Baseline clinical, demographic, and angiographic features and medications are listed in Table 1. Compared to patients with good CC (*n* = 849), those with poor CC (*n* = 709) were older and had higher white blood cells, neutrophils, neutrophil‐to‐lymphocyte ratio, HbA1c, total cholesterol and uric acid but lower left ventricular ejection fraction, eGFR, lymphocyte, and albumin. Renal insufficiency was more prevalent in patients with poor CC (*p* = 0.004). The two groups did not significantly differ with respect to smoking, hypertension, triglyceride, LDL‐C, HDL‐C, severity of coronary artery disease, and medications.

**Table 1:** Baseline clinical characteristics of patients with poor and good CC. The most clinically relevant differences were observed in age, LVEF, HbA1c, and UAR between groups.

**Variables**	**Poor CC (** **n** = 709**)**	**Good CC (** **n** = 849**)**	***p* value**
Age, years	66.0 ± 10.4	63.0 ± 11.1	< 0.001
Female, no. (%)	163 (23.0)	165 (19.4)	0.092
BMI, kg/m^2^	25.17 ± 3.27	25.03 ± 3.36	0.390
SBP, mmHg	137.5 ± 21.7	137.0 ± 19.7	0.638
DBP, mmHg	77.3 ± 12.6	77.7 ± 12.1	0.545
LVEF, %	56.6 ± 9.5	58.6 ± 10.4	< 0.001
Smoking, no. (%)	268 (37.8)	306 (36.0)	0.493
Hypertension, no. (%)	526 (74.2)	601 (70.8)	0.140
Hypercholesterolemia, no. (%)	182 (25.7)	190 (22.4)	0.136
Prior MI, no. (%)	141 (19.9)	142 (16.7)	0.113
Platelet, 10^9^/L	185.0 (157.0–219.0)	181.6 (150.0–219.0)	0.107
White blood cell count, 10^9^/L	7.47 (6.29–9.05)	6.84 (5.70–8.21)	< 0.001
Neutrophil, 10^9^/L	5.40 (4.34–6.96)	4.55 (3.61–5.85)	< 0.001
Lymphocyte, 10^9^/L	1.53 (1.15–1.96)	1.75 (1.42–2.13)	< 0.001
NLR	3.42 (2.74–5.01)	2.78 (1.96–3.47)	< 0.001
Monocyte, 10^9^/L	0.51 (0.40–0.65)	0.50 (0.40–0.63)	0.193
Fasting glucose, mmol/L	6.50 (5.50–7.95)	6.31 (5.24–7.71)	0.062
HbA1c, %	7.00 (6.30–8.10)	6.90 (6.20–7.80)	0.002
Triglyceride, mmol/L	1.56 (1.09–2.11)	1.44 (1.07–2.00)	0.132
Total cholesterol, mmol/L	4.03 (3.29–4.89)	3.89 (3.19–4.69)	0.018
LDL‐C, mmol/L	2.30 (1.70–3.05)	2.20 (1.68–2.92)	0.064
HDL‐C, mmol/L	0.97 (0.85–1.13)	0.97 (0.83–1.13)	0.683
hsCRP, mg/L	2.90 (1.02–6.91)	2.68 (0.91–6.80)	0.119
BUN, mmol/L	6.00 (4.90–7.20)	5.80 (4.70–6.90)	0.188
AST, U/L	23.3 ± 7.8	23.1 ± 8.5	0.569
ALT, U/L	21.7 ± 7.2	21.7 ± 7.2	0.872
Creatinine, *μ*mol/L	82.0 (70.0–96.0)	80.7 (68.0–93.0)	0.066
eGFR, mL/min/1.73 m^2^	78.17 (61.75–94.72)	85.19 (69.25–98.11)	0.001
Renal insufficiency, no. (%)	478 (67.4)	511 (60.2)	0.004
Uric acid, *μ*mol/L	346.0 (294.0–409.0)	292.0 (250.05–337.0)	< 0.001
Albumin, g/L	36.0 (31.0–39.0)	38.0 (35.0–41.0)	< 0.001
UAR	9.74 (8.53–11.95)	7.74 (6.61–8.84)	< 0.001
Severity of CAD, no. (%)			
One‐vessel disease	79(14.0)	75 (10.7)	0.084
Two‐vessel disease	155 (27.4)	185 (26.3)	0.702
Three‐vessel disease	330 (58.3)	448 (63.6)	0.056
Medications, no. (%)			
ACEI/ARB	323 (57.1)	388 (55.1)	0.495
*β*‐Blocker	406 (71.7)	475 (67.5)	0.111
Calcium channel blocker	180 (31.8)	215 (30.5)	0.670
Nitrates	231 (40.8)	303 (43.0)	0.457
Diuretics	117 (20.7)	120 (17.0)	0.111
Statins	504 (89.0)	624 (88.6)	0.858

Abbreviations: ACEI, angiotensin‐converting enzyme inhibitor; ALT, alanine aminotransferase; ARB, angiotensin‐receptor blocker; AST, aspartate aminotransferase; BMI, body mass index; BUN, blood urea nitrogen; CAD, coronary artery disease; CC, coronary collateralization; DBP, diastolic blood pressure; eGFR, estimated glomerular filtration rate; HbA1c, glycosylated hemoglobin A1c; HDL‐C, high‐density lipoprotein cholesterol; hsCRP, high‐sensitivity C‐reactive protein; LDL‐C, low‐density lipoprotein cholesterol; LVEF, left ventricular ejection fraction; MI, myocardial infarction; NLR, neutrophil‐to‐lymphocyte ratio; SBP, systolic blood pressure; UAR, uric acid/albumin ratio.

### 3.2. Uric Acid, Albumin, and UAR With Coronary Collaterals

Serum levels of uric acid decreased (Spearman^’^s *r* = −0.367, *p* < 0.001) and albumin increased (Spearman^’^s *r* = 0.268, *p* < 0.001) gradually across Rentrop score 0 to 3 (Figure 2), resulting in significantly higher UAR in patients with poor collaterals compared to those with good collaterals (9.74 [8.53–11.95] vs. 7.74 [6.61–8.84], *p* < 0.001) (Table 1). Univariate logistic regression analysis revealed that age, left ventricular ejection fraction, neutrophil‐to‐lymphocyte ratio, HbA1c, total cholesterol, eGFR, uric acid, albumin, and UAR were associated with coronary collateral development. After eliminating uric acid and albumin, the collinearity diagnosis showed that age, left ventricular ejection fraction, neutrophil‐to‐lymphocyte ratio, total cholesterol, eGFR, and UAR presented a tolerance over 0.1 and a variance inflation factor under 10, suggesting that no multicollinearity existed among these variables. Multivariate logistic regression models showed that neutrophil–lymphocyte ratio (adjusted OR 1.611, 95% CI 1.474–1.761, *p* < 0.001), HbA1c (adjusted OR 1.242, 95% CI 1.129–1.367, *p* < 0.001) and UAR (adjusted OR 1.887, 95% CI 1.746–2.040, *p* < 0.001) were independent factors for poor CC (Table 2).

Figure 2Distribution of (a) uric acid, (b) albumin, and (c) UAR according to Rentrop score. Uric acid and UAR decreased, whereas albumin increased with higher Rentrop grades. ∗∗∗*p* < 0.001, Spearman correlation; UAR, uric acid‐to‐albumin ratio.

**Figure:**
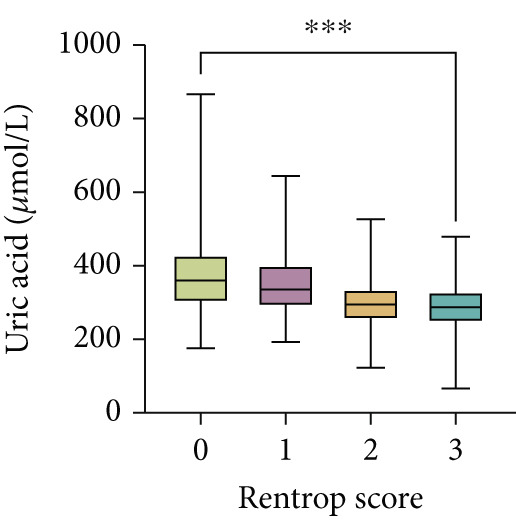
(a)

**Figure:**
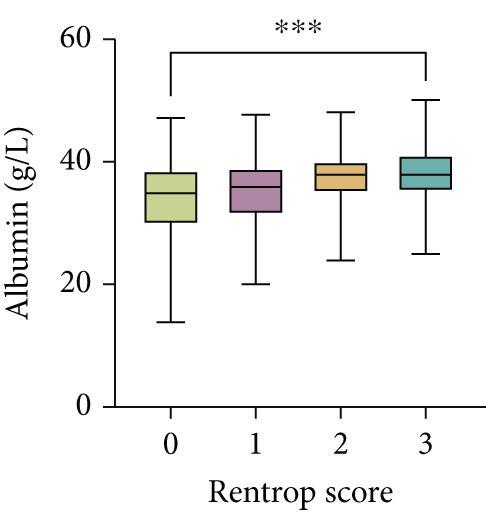
(b)

**Figure:**
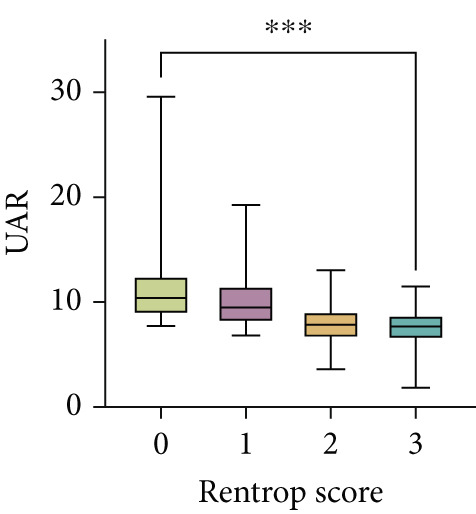
(c)

**Table 2:** Logistic regression analysis of variables for predicting poor CC.

**Univariate analysis**			**Multivariate analysis**	
**Variables**	**OR (95% CI)**	***p* value**	**OR (95% CI)**	***p* value**
Age	1.027 (1.017–1.037)	< 0.001		
LVEF	0.981 (0.972–0.991)	< 0.001		
NLR	1.562 (1.450–1.683)	< 0.001	1.611 (1.474–1.761)	< 0.001
HbA1c	1.153 (1.072–1.239)	< 0.001	1.242 (1.129–1.367)	< 0.001
Total cholesterol	1.122 (1.032–1.220)	0.007		
eGFR	0.985 (0.980–0.989)	< 0.001		
Uric acid	1.010 (1.009–1.012)	< 0.001		
Albumin	0.888 (0.869–0.907)	< 0.001		
UAR	1.745 (1.634–1.863)	< 0.001	1.887 (1.746–2.040)	< 0.001

Abbreviations: CI, confidence interval; CC, coronary collateralization; eGFR, estimated glomerular filtration rate; HbA1c, glycosylated hemoglobin A1c; LVEF, left ventricular ejection fraction; NLR, neutrophil‐to‐lymphocyte ratio; OR, odds ratio; UAR, uric acid‐to‐albumin ratio.

**Figure 3:**
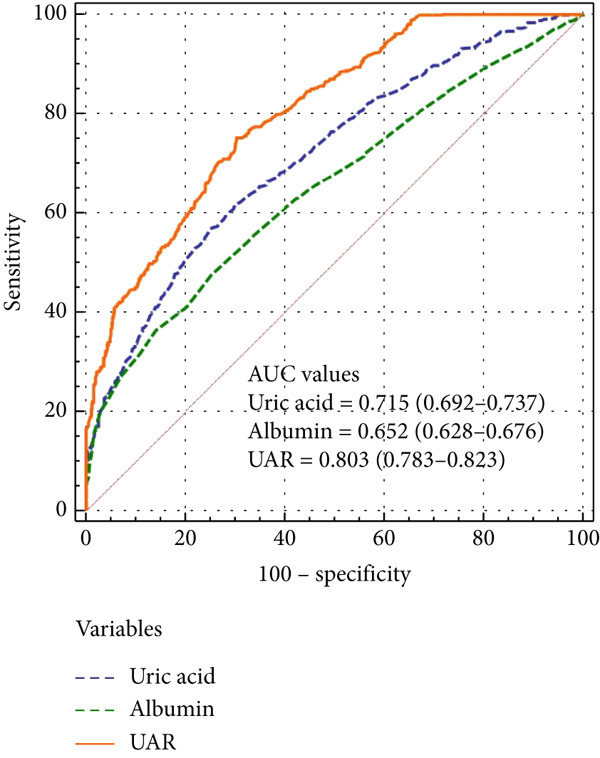
Receiver operating characteristic (ROC) curves for predicting poor coronary collateralization using uric acid‐to‐albumin ratio (UAR), uric acid, and albumin. According to the ROC analysis, UAR demonstrated superior discriminative ability compared with uric acid and albumin.

In ROC curve analysis, we identified 8.5 as the optimal UAR cut off value using the Youden index. This threshold demonstrated good discriminatory ability for differentiate patients with poor CC from good CC, with an AUC of 0.803 (95% CI 0.783–0.823), sensitivity of 75.18%, and specificity of 69.73%. UAR had a significantly better predictive ability for poor CC than uric acid (AUC 0.715, 95% CI 0.692–0.737) and albumin (AUC 0.652, 95% CI 0.628–0.676) (all *p* < 0.001) (Figure 3).

### 3.3. Influence of Renal Function

**Figure 5:**
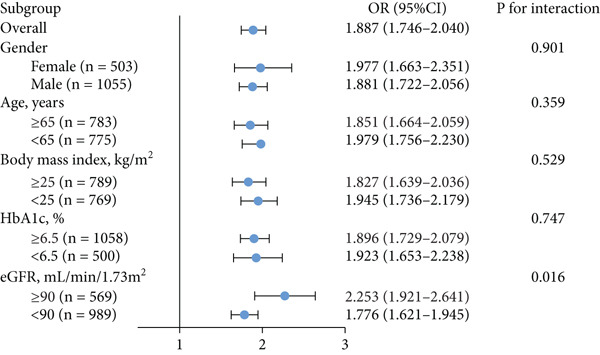
Diagnostic value of UAR in patient subgroup analysis. HbA1c, glycosylated hemoglobin A1c; eGFR, estimated glomerular filtration rate; OR, odds ratio; CI, confidence interval.

In total, preserved renal function and mild, moderate, or severe renal insufficiency were detected in 569, 723, 255, and 11 patients, respectively. Compared to those with preserved renal function, the occurrence of UAR ≥ 8.5 and poor CC was higher in patients with renal insufficiency, which became more obvious with the progression of renal impairment (Figure 4). There existed an interaction between UAR and eGFR (*p* = 0.016), and elevated UAR was associated with a greater risk for poor CC in patients with preserved renal function compared to those with renal insufficiency (OR 2.253 vs. 1.776) (Figure 5). In addition, UAR provided a higher predictive value for poor CC in patients with preserved renal function compared to those with renal impairment (AUC 0.830 vs. 0.786, *p* = 0.041).

Figure 4Comparison between patients with different renal functional status ∗*p* < 0.05, ∗∗∗*p* < 0.001. CC, coronary collateralization; UAR, uric acid/albumin ratio; eGFR, estimated glomerular filtration rate.

**Figure:**
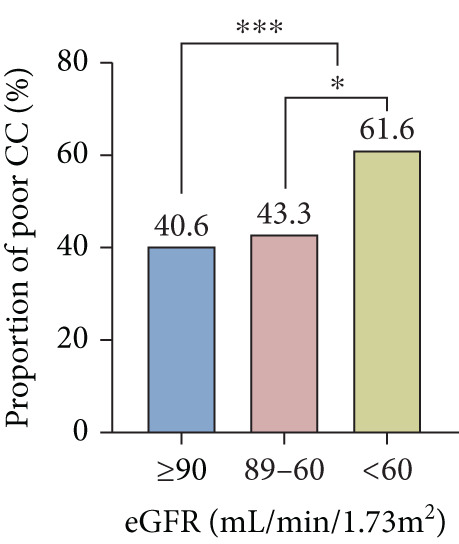
(a)

**Figure:**
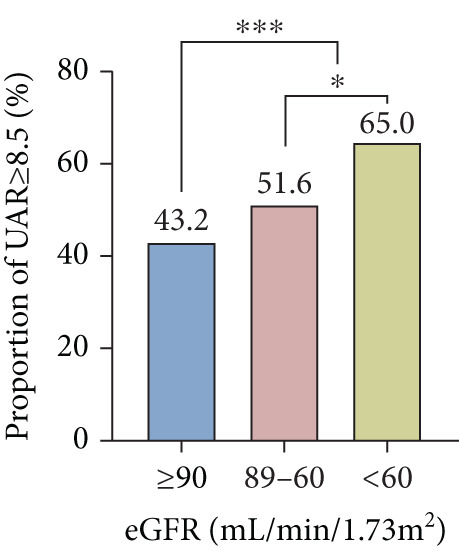
(b)

## 4. Discussion

The results of this study show that in type 2 diabetic patients with stable coronary artery disease, elevated UAR was closely associated with reduced collateral formation, which was superior to serum uric acid and albumin alone. Its predictive ability for poor CC was significantly better in patients with preserved renal function compared to those with renal insufficiency. To our knowledge, few studies have directly investigated the relationship between UAR and coronary collateral development [13, 14]. Our findings are consistent with these results and further confirm the value of UAR in a diabetic population, especially those with preserved renal function. Importantly, we identified a significant interaction between renal function and the predictive value of UAR, suggesting that renal insufficiency may attenuate its utility as a biomarker. It is well recognized that diabetes impairs collateral vessel growth and maturation through multiple mechanisms involving arteriogenesis and angiogenesis, influenced by various clinical, biochemical, and angiographic factors [4, 21]. Persistent low‐grade inflammation in diabetic patients promotes oxidative stress and endothelial dysfunction, which negatively affect collateral development [22]. In line with previous reports [23–26], we found that neutrophil‐to‐lymphocyte ratio (NLR), a classic inflammatory index, was independently associated with poor CC. Likewise, HbA1c, a most widely used parameter for optimal glycemic control and management of diabetes, has been shown to correlate closely with the occurrence of macro‐ and micro‐vascular complications [27–29]. Mechanistically, advanced glycation end‐products and their receptors (AGE‐RAGE axis) activate nicotinamide adenine dinucleotide phosphate oxidase (NOX) and uncouple eNOS, leading to excessive ROS production and impaired endothelial function [30]. We observed that increased HbA1c was an independent factor of reduced coronary collaterals in T2DM patients with CTO.

The present study further demonstrated that elevated UAR was independently associated with poor CC, and its predictive value was superior to serum uric acid and albumin alone. This is likely due to the synergistic impact of high uric acid and low albumin levels on vascular health. Hyperuricemia activates the renin–angiotensin system and inhibits endothelial nitric oxide production, all contributing to vascular injury [31]. Conversely, hypoalbuminemia impairs clearance of reactive oxygen species and disrupts circulatory homeostasis [32], further hampering collateral vessel growth [33, 34]. Moreover, increased arterial stiffness, correlated with higher uric acid and lower albumin levels [35], reduces coronary perfusion and shear stress, negatively influencing collateral development [36].

In our study, the UAR was independently associated with poor CC with an odds ratio (OR) of 1.887, indicating that each unit increase in UAR increased the likelihood of poor CC by approximately 89%. Furthermore, the area under the ROC curve (AUC) for UAR was 0.803, suggesting good discriminatory ability. Clinically, UAR could be incorporated into existing risk stratification tools for T2DM patients with CTO, helping identify high‐risk individuals who may benefit from more aggressive secondary prevention or closer monitoring prior to revascularization. Compared with other emerging inflammatory or oxidative stress related biomarkers, such as NLR or systemic immune‐inflammation index, UAR offers a simple, inexpensive, and readily obtainable index that reflects both oxidative stress and inflammatory status. Although UAR alone may not be sufficient for decision‐making, its relatively high AUC suggests that it could complement existing models to improve early identification of high‐risk patients and refine individualized management strategies.

In addition, our results show that the predictive value of UAR was significant in patients with preserved renal function but attenuated in those with renal insufficiency. The mechanism underlying this phenomenon remains unclear but may involve several interrelated biological pathways. Previous studies have indicated that in diabetic individuals, an increased inflammatory response combined with hyperuricemia significantly raises the risk of chronic kidney disease, and renal impairment worsens as diabetes progresses through multiple pathways and mediators [37, 38]. Clinical studies have demonstrated that elevated circulating inflammatory cytokines are associated with reduced coronary collateral growth. Xie et al. found that coronary collateral formation is reduced even in the presence of mild renal insufficiency [39]. Moreover, chronic hypoxia in the kidney has been recognized as a key contributor to kidney injury. Several hypoxia‐induced proteins such as hypoxia‐inducible factor and vascular endothelial growth factor are involved in angiogenesis and associated with coronary collateral development. However, these adaptive pathways are suppressed in renal insufficiency due to aggregated oxidative stress. Renal insufficiency is characterized by systemic endothelial dysfunction, which adversely affects several processes essential for collateral formation, including angiogenesis, vascular remodeling, and endothelial proliferation. These pervasive alterations in vascular homeostasis may overshadow or mask the specific contribution of the UAR. In addition, disturbances in mineral metabolism, anemia, and volume overload commonly seen in renal dysfunction further impair endothelial repair and microvascular adaptation. As a result, in advanced renal disease, the incremental prognostic value of UAR may be diminished in the setting of multiple vascular injuries.

## 5. Limitations

Our study has certain limitations. First, as a cross‐sectional study, we could only detect an association but not a causal link between UAR and coronary collaterals. Therefore, causality cannot be inferred. Future prospective and longitudinal studies with long‐term follow‐up are warranted to validate these findings and further clarify the prognostic value of UAR. Second, although the use of a large, consecutively enrolled cohort and standardized data collection enhances the representativeness of our findings, certain lifestyle or behavioral factors such as dietary habits, physical activity levels, or medication adherence were not measured in this study and may have influenced the results. Future studies incorporating detailed lifestyle assessments are needed to address this limitation. Additionally, 147 patients were excluded due to missing clinical or angiographic data, because of the incomplete data available for these patients, we were unable to conduct a detailed comparison with the included cohort. This limitation may introduce potential selection bias and affect our ability to evaluate the direction and magnitude of its impact on the study findings. Third, the Rentrop scoring system used to assess coronary collaterals is subject to observer variability and is less precise. We chose this approach because it is noninvasive, easily applicable in retrospective angiographic analyses, and provides a practical estimate of collateral development in large patient cohorts. Nevertheless, more objective techniques such as the collateral flow index or other invasive hemodynamic assessments may offer greater accuracy and should be considered in future studies. Fourth, while we discussed possible mechanisms linking UAR and collateral development, these were not directly investigated.

## 6. Conclusions

This study is the first to demonstrate that elevated UAR is associated with poor CC in T2DM patients with CTO and provides a superior predictive ability than uric acid and albumin alone, particularly in those with preserve renal function. Owing to the important clinical implications of coronary collateral circulation, the predictive value of UAR on coronary collateral formation could assist physicians to make appropriate clinical decisions. Future prospective and interventional studies, as well as validation in multi‐center cohorts, are warranted to determine if the association between elevated UAR and poor angiographic coronary collaterals could be translated into incremental therapeutic and prognostic information in type 2 diabetic patients with coronary artery disease.

## Ethics Statement

The study protocol was approved by the Institutional Review Board of Rui Jin Hospital, Shanghai Jiao Tong University School of Medicine. Written informed consent was obtained from all patients, and clinical investigation was conducted according to the principle of the Declaration of Helsinki.

## Disclosure

All authors read and approved the final manuscript.

## Conflicts of Interest

The authors declare no conflicts of interest.

## Author Contributions

L.S.M. and Y.S. contributed to the conceptualization. L.S.M., Y.X.W., Y.Q., and Y.S. contributed to the methodology. L.S.M., Y.X.W., Y.Q., and Z.R.Y. contributed to the investigation and data curation. L.S.M. and F.H.D. performed data analysis. X.Q.W., L.L., W.F.S., and Y.S. contributed to the resources. L.S.M. performed visualizations. W.F.S. and Y.S. supervised the study. Y.S. contributed to the funding acquisitions. L.S.M. contributed to the writing—original draft. L.S.M. and Y.S. contributed to the writing—review and editing.

## Funding

This study is supported by the National Natural Science Foundation of China, 10.13039/501100001809, 82370409, 82170417.

## Data Availability

The datasets used or analyzed during the current study are available from the corresponding author on reasonable request. The analytic code used for data processing and statistical analysis can also be provided upon request to ensure reproducibility.

## References

[1] Yang Z. K. , Shen Y. , Dai Y. , Wang X. Q. , Hu J. , Ding F. H. , Zhang R. Y. , Lu L. , and Shen W. F. , Impact of Coronary Collateralization on Long-Term Clinical Outcomes in Type 2 Diabetic Patients After Successful Recanalization of Chronic Total Occlusion, Cardiovascular Diabetology. (2020) 19, no. 1, 10.1186/s12933-020-01033-4, 32393276. PMC7216347 32393276

[2] Jamaiyar A. , Juguilon C. , Dong F. , Cumpston D. , Enrick M. , Chilian W. M. , and Yin L. , Cardioprotection During Ischemia by Coronary Collateral Growth, American Journal of Physiology. Heart and Circulatory Physiology. (2019) 316, no. 1, H1–h9, 10.1152/ajpheart.00145.2018, 2-s2.0-85060252644. 30379567 PMC6383359

[3] Allahwala U. K. , Khachigian L. M. , Nour D. , Ridiandres A. , Billah M. , Ward M. , Weaver J. , and Bhindi R. , Recruitment and Maturation of the Coronary Collateral Circulation: Current Understanding and Perspectives in Arteriogenesis, Microvascular Research. (2020) 132, 104058, 10.1016/j.mvr.2020.104058, 32798552. 32798552

[4] Shen Y. , Ding F. H. , Dai Y. , Wang X. Q. , Zhang R. Y. , Lu L. , and Shen W. F. , Reduced Coronary Collateralization in Type 2 Diabetic Patients With Chronic Total Occlusion, Cardiovascular Diabetology. (2018) 17, no. 1, 10.1186/s12933-018-0671-6, 2-s2.0-85041842974, 29422093. PMC5804044 29422093

[5] Wu Z. M. , Huang K. , Dai Y. , Chen S. , Wang X. Q. , Yang C. D. , Li L. Y. , Liu J. M. , Lu L. , Zhang R. Y. , Shen W. F. , Shen Y. , and Ding F. H. , Circulating Secretoneurin Level Reflects Angiographic Coronary Collateralization in Stable Angina Patients With Chronic Total Occlusion, BMC Cardiovascular Disorders. (2024) 24, no. 1, 10.1186/s12872-023-03645-6, 38184555. PMC10771680 38184555

[6] Shen Y. , Wang X. Q. , Dai Y. , Wang Y. X. , Zhang R. Y. , Lu L. , Ding F. H. , and Shen W. F. , Diabetic Dyslipidemia Impairs Coronary Collateral Formation: An Update, Frontiers in Cardiovascular Medicine. (2022) 9, 956086, 10.3389/fcvm.2022.956086, 36072863. 36072863 PMC9441638

[7] Saito Y. , Tanaka A. , Node K. , and Kobayashi Y. , Uric Acid and Cardiovascular Disease: A Clinical Review, Journal of Cardiology. (2021) 78, no. 1, 51–57, 10.1016/j.jjcc.2020.12.013. 33388217

[8] Manolis A. A. , Manolis T. A. , Melita H. , Mikhailidis D. P. , and Manolis A. S. , Low Serum Albumin: A Neglected Predictor in Patients With Cardiovascular Disease, European Journal of Internal Medicine. (2022) 102, 24–39, 10.1016/j.ejim.2022.05.004, 35537999. 35537999

[9] Arques S. , Serum Albumin and Cardiovascular Disease: State-of-the-Art Review, Annales de Cardiologie et d′Angéiologie. (2020) 69, no. 4, 192–200, 10.1016/j.ancard.2020.07.012. 32797938

[10] Chen S. , Zhang M. , Hu S. , Shao X. , Liu L. , Yang Z. , and Nan K. , Uric Acid to Albumin Ratio Is a Novel Predictive Marker for All-Cause and Cardiovascular Death in Diabetic Patients: A Prospective Cohort Study, Frontiers in Endocrinology. (2024) 15, 1388731, 10.3389/fendo.2024.1388731. 39911231 PMC11794066

[11] Çakmak E. , Bayam E. , Çelik M. , Kahyaoğlu M. , Eren K. , Imanov E. , Karagöz A. , and İzgi İ. A. , Uric Acid-to-Albumin Ratio: A Novel Marker for the Extent of Coronary Artery Disease in Patients With Non-ST-Elevated Myocardial Infarction, Pulse. (2021) 8, no. 3-4, 99–107, 10.1159/000514533, 34307206. 34307206 PMC8280454

[12] Kalkan S. , Cagan Efe S. , Karagöz A. , Zeren G. , Yılmaz M. F. , Şimşek B. , Batgerel U. , Özkalaycı F. , Tanboğa İ. H. , Oduncu V. , Karabay C. Y. , and Kırma C. , A New Predictor of Mortality in ST-Elevation Myocardial Infarction: The Uric Acid Albumin Ratio, Angiology. (2022) 73, no. 5, 461–469, 10.1177/00033197211066362, 34989646. 34989646

[13] Yin R. , Ye Z. , You H. , Wu Y. , Chen W. , and Jiang T. , Elevated Uric Acid/Albumin Ratio as a Predictor of Poor Coronary Collateral Circulation Development in Patients With Non-ST Segment Elevation Myocardial Infarction, Clinical Cardiology. (2024) 47, no. 1, e24215, 10.1002/clc.24215. 38269629 PMC10790324

[14] Şaylık F. , Çınar T. , Sarıkaya R. , Akbulut T. , Selçuk M. , Özbek E. , and Tanboğa H. İ. , The Association of Serum Uric Acid/Albumin Ratio With the Development of Coronary Collateral Circulation in Patients With Chronic Total Occluded Coronary Arteries, Journal of Cardiovascular and Thoracic Research. (2023) 15, no. 1, 14–21, 10.34172/jcvtr.2023.31627, 37342660. 37342660 PMC10278190

[15] Levey A. S. , Stevens L. A. , Schmid C. H. , Zhang Y. ( L.) , Castro A. F.III, Feldman H. I. , Kusek J. W. , Eggers P. , van Lente F. , Greene T. , Coresh J. , and for the CKD-EPI (Chronic Kidney Disease Epidemiology Collaboration) , A New Equation to Estimate Glomerular Filtration Rate, Annals of Internal Medicine. (2009) 150, no. 9, 604–612, 10.7326/0003-4819-150-9-200905050-00006, 2-s2.0-65649142017. 19414839 PMC2763564

[16] Wang X. , Jiang L. , and Shao X. , Association Analysis of Insulin Resistance and Osteoporosis Risk in Chinese Patients With T2DM, Therapeutics and Clinical Risk Management. (2021) Volume 17, 909–916, 10.2147/TCRM.S328510, 34511917. 34511917 PMC8418372

[17] Naganawa H. , Ito A. , Saiki S. , Nishi D. , Takamatsu S. , Ito Y. , and Suzuki T. , The Efficacy of Drug-Coated Balloon for Acute Coronary Syndrome, Cardiology Research and Practice. (2023) 2023, 4594818, 10.1155/2023/4594818. 37122873 PMC10139813

[18] Kolland M. , Hofer E. , Pirpamer L. , Eibl D. , Enzinger C. , Rosenkranz A. R. , and Schmidt R. , Kidney Function, Brain Morphology and Cognition in the Elderly: Sex Differences in the Austrian Stroke Prevention Study, Aging. (2022) 14, no. 1, 240–252, 10.18632/aging.203829, 35025758. 35025758 PMC8791200

[19] Guo X. , Cao J. , Liu P. , Cao Y. , Li X. , Gao L. , Wang Z. , Fang L. , Jin Z. , Wang Y. , and Xing B. , Cardiac Abnormalities in Acromegaly Patients: A Cardiac Magnetic Resonance Study, International Journal of Endocrinology. (2020) 2020, 2018464, 10.1155/2020/2018464, 32148485. 32148485 PMC7042537

[20] Rentrop K. P. , Cohen M. , Blanke H. , and Phillips R. A. , Changes in Collateral Channel Filling Immediately After Controlled Coronary Artery Occlusion by an Angioplasty Balloon in Human Subjects, Journal of the American College of Cardiology. (1985) 5, no. 3, 587–592, 10.1016/S0735-1097(85)80380-6, 2-s2.0-0021923120. 3156171

[21] Rocic P. , Why Is Coronary Collateral Growth Impaired in Type II Diabetes and the Metabolic Syndrome?, Vascular Pharmacology. (2012) 57, no. 5-6, 179–186, 10.1016/j.vph.2012.02.001, 2-s2.0-84865658925, 22342811. 22342811 PMC3359422

[22] Weinberg Sibony R. , Segev O. , Dor S. , and Raz I. , Overview of Oxidative Stress and Inflammation in Diabetes, Journal of Diabetes. (2024) 16, no. 10, e70014, 10.1111/1753-0407.70014. 39435991 PMC11494684

[23] Mao L. S. , Wang Y. X. , Wu Z. M. , Ding F. H. , Lu L. , Shen W. F. , Dai Y. , and Shen Y. , Elevated Systemic Immune-Inflammatory Index Predicts Poor Coronary Collateralization in Type 2 Diabetic Patients With Chronic Total Occlusion, Frontiers in Cardiovascular Medicine. (2024) 11, 1490498, 10.3389/fcvm.2024.1490498, 39735863. 39735863 PMC11672344

[24] Nacar A. B. , Erayman A. , Kurt M. , Buyukkaya E. , Karakaş M. F. , Akcay A. B. , Buyukkaya S. , and Sen N. , The Relationship Between Coronary Collateral Circulation and Neutrophil/Lymphocyte Ratio in Patients With Coronary Chronic Total Occlusion, Medical Principles and Practice. (2015) 24, no. 1, 65–69, 10.1159/000365734, 2-s2.0-84921439073, 25342010. 25342010 PMC5588179

[25] Açar G. , Kalkan M. E. , Avci A. , Alizade E. , Tabakci M. M. , Toprak C. , Özkan B. , Alici G. , and Esen A. M. , The Relation of Platelet-Lymphocyte Ratio and Coronary Collateral Circulation in Patients With Stable Angina Pectoris and Chronic Total Occlusion, Clinical and Applied Thrombosis/Hemostasis. (2015) 21, no. 5, 462–468, 10.1177/1076029613508599, 2-s2.0-84933034054, 24142833. 24142833

[26] Fan Y. , Li S. , Li X. L. , Zhu C. G. , Guo Y. L. , Wu N. Q. , Qing P. , Gao Y. , Dong Q. , Liu G. , and Li J. J. , C-Reactive Protein as a Predictor for Poor Collateral Circulation in Patients With Chronic Stable Coronary Heart Disease, Annals of Medicine. (2016) 48, no. 1-2, 83–88, 10.3109/07853890.2015.1136429, 2-s2.0-84958750327, 26790524. 26790524

[27] Kiss L. Z. , Bagyura Z. , Vadas R. , Polgár L. , Lux Á. , Édes E. , Szenczi O. , Soós P. , Szelid Z. , Becker D. , Jermendy G. , and Merkely B. , Signs of Subclinical Atherosclerosis in Asymptomatic Patients at Increased Risk of Type 2 Diabetes Mellitus, Journal of Diabetes and its Complications. (2017) 31, no. 8, 1293–1298, 10.1016/j.jdiacomp.2017.05.007, 2-s2.0-85020052550, 28576484. 28576484

[28] Santos R. D. , Shapiro M. D. , and Ballantyne C. M. , Glycated Hemoglobin to Detect Subclinical Atherosclerosis in People Without Diabetes, Journal of the American College of Cardiology. (2021) 77, no. 22, 2792–2795, 10.1016/j.jacc.2021.04.018, 34082908. 34082908

[29] Shen Y. , Lu L. , Ding F. H. , Sun Z. , Zhang R. , Zhang Q. , Yang Z. , Hu J. , Chen Q. , and Shen W. , Association of Increased Serum Glycated Albumin Levels With Low Coronary Collateralization in Type 2 Diabetic Patients With Stable Angina and Chronic Total Occlusion, Cardiovascular Diabetology. (2013) 12, no. 1, 10.1186/1475-2840-12-165, 2-s2.0-84887360982. PMC4225762 24209601

[30] Meza C. A. , La Favor J. D. , Kim D. H. , and Hickner R. C. , Endothelial Dysfunction: Is There a Hyperglycemia-Induced Imbalance of NOX and NOS?, International Journal of Molecular Sciences. (2019) 20, no. 15, 10.3390/ijms20153775, 2-s2.0-85071023719, 31382355. PMC6696313 31382355

[31] Ko J. , Kang H. J. , Kim D. A. , Kim M. J. , Ryu E. S. , Lee S. , Ryu J. H. , Roncal C. , Johnson R. J. , and Kang D. H. , Uric Acid Induced the Phenotype Transition of Vascular Endothelial Cells via Induction of Oxidative Stress and Glycocalyx Shedding, FASEB Journal. (2019) 33, no. 12, 13334–13345, 10.1096/fj.201901148R. 31553887

[32] Pignatelli P. , Farcomeni A. , Menichelli D. , Pastori D. , and Violi F. , Serum Albumin and Risk of Cardiovascular Events in Primary and Secondary Prevention: A Systematic Review of Observational Studies and Bayesian Meta-Regression Analysis, Internal and Emergency Medicine. (2020) 15, no. 1, 135–143, 10.1007/s11739-019-02204-2. 31605272

[33] Zhao Y. , Wang S. , Yang J. , Lin Z. , and Chen Q. , Association of Fibrinogen/Albumin Ratio and Coronary Collateral Circulation in Stable Coronary Artery Disease Patients, Biomarkers in Medicine. (2020) 14, no. 16, 1513–1520, 10.2217/bmm-2020-0333, 33200965. 33200965

[34] Kelesoglu S. , Yilmaz Y. , and Elcık D. , Relationship Between C-Reactive Protein to Albumin Ratio and Coronary Collateral Circulation in Patients With Stable Coronary Artery Disease, Angiology. (2021) 72, no. 9, 829–835, 10.1177/00033197211004392, 33759588. 33759588

[35] Zhang J. , Xiang G. , Xiang L. , and Sun H. , Serum Uric Acid Is Associated With Arterial Stiffness in Men With Newly Diagnosed Type 2 Diabetes Mellitus, Journal of Endocrinological Investigation. (2014) 37, no. 5, 441–447, 10.1007/s40618-013-0034-9, 2-s2.0-84900546956, 24682912. 24682912

[36] Baykan A. O. , Gür M. , Acele A. , Şeker T. , Quisi A. , Yildirim A. , and Çayli M. , Coronary Collateral Development and Arterial Stiffness in Patients With Chronic Coronary Total Occlusions, Scandinavian Cardiovascular Journal. (2015) 49, no. 4, 228–234, 10.3109/14017431.2015.1062130, 2-s2.0-84936743415, 26073524. 26073524

[37] Samsu N. , Diabetic Nephropathy: Challenges in Pathogenesis, Diagnosis, and Treatment, BioMed Research International. (2021) 2021, 1497449, 10.1155/2021/1497449. 34307650 PMC8285185

[38] Ma L. , Wang J. , Ma L. , and Wang X. M. , The Link Between Hyperuricemia and Diabetes: Insights From a Quantitative Analysis of Scientific Literature, Frontiers in Endocrinology. (2024) 15, 1441503, 10.3389/fendo.2024.1441503. 39991045 PMC11842261

[39] Xie S. L. , Li H. Y. , Deng B. Q. , Luo N. S. , Geng D. F. , Wang J. F. , and Nie R. Q. , Poor Coronary Collateral Vessel Development in Patients With Mild to Moderate Renal Insufficiency, Clinical Research in Cardiology. (2011) 100, no. 3, 227–233, 10.1007/s00392-010-0233-8, 2-s2.0-79954570684, 20865265. 20865265

